# The role of club cell phenoconversion and migration in idiopathic pulmonary fibrosis

**DOI:** 10.18632/aging.101115

**Published:** 2016-11-29

**Authors:** Jutaro Fukumoto, Ramani Soundararajan, Joseph Leung, Ruan Cox, Sanjay Mahendrasah, Neha Muthavarapu, Travis Herrin, Alexander Czachor, Lee C. Tan, Nima Hosseinian, Priyanshi Patel, Jayanthraj Gone, Mason T. Breitzig, Young Cho, Andrew J. Cooke, Lakshmi Galam, Venkata Ramireddy Narala, Yashwant Pathak, Richard F. Lockey, Narasaiah Kolliputi

**Affiliations:** ^1^ Division of Allergy and Immunology, Department of Internal Medicine, Morsani College of Medicine, University of South Florida, Tampa, FL 33612, USA; ^2^ Department of Molecular Medicine, Morsani College of Medicine, University of South Florida, Tampa, FL 33612, USA; ^3^ College of Pharmacy, University of South Florida, Tampa, FL 33612, USA; ^4^ Department of Zoology, Yogi Vemana University, Kadapa, AP 516003 India

**Keywords:** club cells, idiopathic pulmonary fibrosis (IPF), Claudin10/Cldn10/Claudin-10, club cell secretory protein (CCSP), migration

## Abstract

Idiopathic pulmonary fibrosis (IPF) is an age-related multifactorial disease featuring non-uniform lung fibrosis. The decisive cellular events at early stages of IPF are poorly understood. While the involvement of club cells in IPF pathogenesis is unclear, their migration has been associated with lung fibrosis. In this study, we labeled club cells immunohistochemically in IPF lungs using a club cell marker Claudin-10 (Cldn10), a unique protein based on the recent report which demonstrated that the appearance of Cldn10 in developing and repairing lungs precedes other club cell markers including club cell secretory protein (CCSP). Cldn10-positive cells in IPF lungs displayed marked pleomorphism and were found in varied arrangements, suggesting their phenoconversion. These results were corroborated by immunogold labeling for Cldn10. Further, immunohistochemical double-labeling for Cldn10 and α-smooth muscle actin (α-SMA) demonstrated that aberrant α-SMA signals are frequently encountered near disorganized Cldn10-positive cells in hyperplastic bronchiolar epithelium and thickened interstitium of IPF lungs. Collectively, these data indicate that club cells actively participate in the initiation and progression of IPF through phenoconversion involving the acquisition of proliferative and migratory abilities. Thus, our new findings open the possibility for club cell-targeted therapy to become a strategic option for the treatment of IPF.

## INTRODUCTION

Idiopathic pulmonary fibrosis (IPF) is an age-related, chronic, and progressive lung disease of unknown etiology [1]. Notably, the key cellular and molecular events in early stage IPF are poorly understood [2]. Recent reports suggest that type II alveolar epithelial cell (AEC) dysfunction, caused by gene mutations, coupled with repetitive exposure to noxious stimuli contributes to IPF development [3,4]. As an example of such genetic predispositions related to pulmonary fibrosis, mutations in SFTPC, a gene encoding surfactant protein C (pro-SPC, a representative marker of type II AECs), have been associated with familial pulmonary fibrosis (FPF) kindreds. Patients with SFTPC mutations present with a histopathological pattern of usual interstitial pneumonia (UIP), a key pathological feature of IPF [5,6]. In the meantime, a particular minor allele of single-nucleotide polymorphism (SNP) in the putative promoter region of MUC5B, a gene largely expressed in bronchiolar epithelium has also been linked to familial interstitial pneumonia and IPF [7]. This indicates that not only type II AEC dysfunction, but also functional perturbation of the bronchiolar epithelial cells is a risk factor for pulmonary fibrosis.

club cells (previously Clara cells) are non-ciliated bronchiolar epithelial cells with multiple functions including (i) xenobiotic metabolism, (ii) immuno-modulation through secretion of club cell secretory protein (CCSP), and (iii) regeneration through progenitor activity [8]. The involvement of club cells in IPF or other lung diseases featuring pulmonary fibrosis is not clear, however, it has been continually suggested since the 1980s that there is a link between lung fibrosis and alveolar bronchiolization, a process where club cells and other bronchiolar epithelial cell types migrate and populate alveolar walls [9–13]. Intriguingly, a recent report provided novel insights into a pathological role for club cells in IPF, wherein the authors proposed that club cells accelerate IPF progression through promoting lung epithelial cell death [13]. Madala et al. demonstrated that club cell-specific overexpression of transforming growth factor alpha (TGF-ɑ) activate mesenchymal cell migration and accumulation in lung fibrosis [14]. In spite of such rising attention of recent years being paid to club cells, the cumulative attention that club cells have garnered so far in the field of IPF is very little when it is compared to type II AECs. One of the reasons attributed to this is the relative sparsity of club cells, as defined and assessed by the expression of CCSP, in IPF lungs in comparison to type II AECs.

In most of the lung fibrosis studies published so far, CCSP expression was used to define and trace club cells. However, a recent study has identified an additional club cell markers [15]. Given the availability of newly established club cell markers, no studies were initiated with these markers to investigate the potential contribution of club cells to IPF pathology. The newly identified club cell markers include, but are not limited to, Flavin monooxygenase 3 (Fmo3), paraoxonase 1 (Pon1), aldehyde oxidase 3 (Aox3) and Claudin-10 (Cldn10). Among these newly identified club cell markers, Claudin-10 (referred to as Cldn10 hereinafter) is a very unique protein. In the early developing lungs of mice, Cldn10 first appears throughout the developing airway epithelium, and as club cells mature and begin to express CCSP, Cldn10 expression converges to the lateral surface of club cells (Supplemental Figure S1A) [15]. This spatial expression pattern of Cldn10 in adult lung club cells is consistent with the fact that Cldn10 have functions in paracellular epithelial permeation as tight junction components. Most importantly, Cldn10's expression in developing lungs appears earlier than all other club cell markers including CCSP. The expression of Cldn10 in mouse lungs reaches adult expression levels as early as embryonic day (E) 17.5, an earlier time-point than CCSP, of which expression at E17.5 is still very low and reaches adult expression levels only as late as postnatal day 7 (P7) (Supplemental Figure S1B) [15]. In addition, the re-establishment of Cldn10 in damaged bronchiolar epithelium after naphthalene-induced club cell ablation occurs more rapidly than CCSP [15]. Thus, Cldn10 is considered to be an early sign for the developing and repairing bronchiolar epithelium. In other words, Cldn10 is a sensitive tracer for immature and not fully differentiated club cells in both developing and repairing airways. Intriguingly, IPF has been repetitively associated with recapitulation of developmental pathways [16,17]. Further, impaired wound healing has been considered to be a key mechanism in the pathogenesis of IPF [18]. Thus, we surmised that pleiotropic evaluation of Cldn10 would shed light on the potential involvement of club cells in IPF pathogenesis in the context of aberrant activation of developmental and repair pathways.

## RESULTS

### Attenuation of CCSP expression in bronchiolar epithelium are associated with interstitial fibrosis and formation of fibroblastic foci in IPF lungs

**Figure 1:**
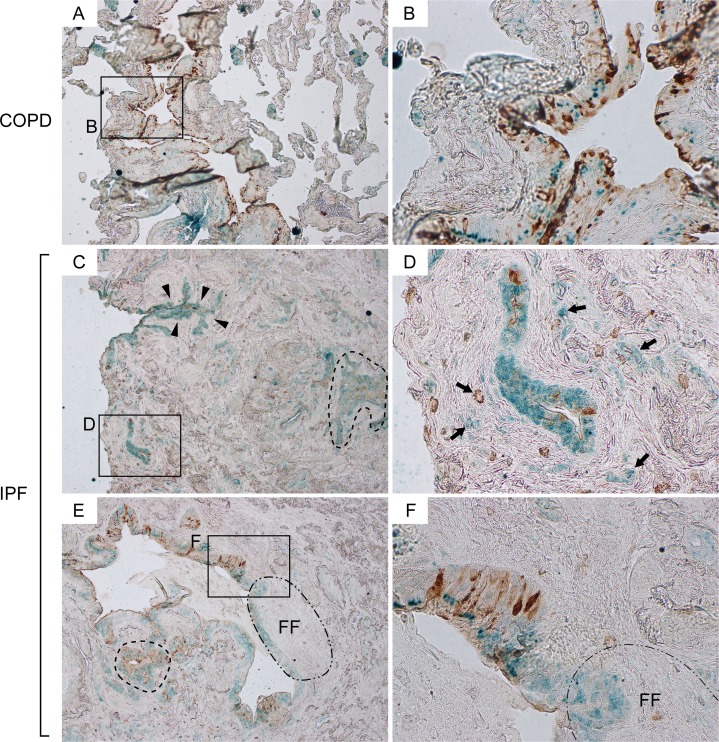
Attenuation of CCSP expression in bronchiolar epithelium are associated with interstitial fibrosis and formation of fibroblastic foci in IPF lungs Paraffin-embedded lung sections from patients with idiopathic pulmonary fibrosis (IPF) and chronic obstructive pulmonary fibrosis (COPD) were immunohistochemically double-labeled for club cell secretory protein (CCSP) and Claudin-10 (Cldn10). Brown and green signals correspond to CCSP and Cldn10 respectively. (**A, B**) Representative photomicrographs from COPD lungs display an area which shows colocalization of the two signals in bronchiolar epithelium (**B**). Solely Cldn10-positive club cells are seen in slightly affected alveoli (**A**). (**C, D**) Photomicrographs of highly fibrotic lesions from IPF lungs are shown. CCSP signals are scarcely scattered. Dashed-line in (**C**) denotes a distinct area with CCSP- and/or Cldn10-positve club cells having occluded a bronchiolar lumen. Arrows in (**D**) denote club cells blended with the surrounding fibrotic interstitium. (**E, F**) Photomicrographs of a widely open bronchiolar lumen and surrounding fibrotic interstitium from IPF lungs are shown. Dashed-line in (**E**) denotes an area with club cell hypercellularity. Area circled by dot-dashed line (FF) denotes fibroblastic foci. Luminal side of fibroblastic foci is lined by CCSP-negative Cldn10-positive club cells (**E**) whereas the adjacent bronchiolar epithelium exhibits both signals (**F**). Original magnifications: x40 (**A, C, E**); x400 (**B, D, F**).

Cldn10 is a recently identified marker for club cells. Given its unique expression pattern in the developing and repairing airways [15], Cldn10 is considered to be a sensitive tracer for immature and not fully differentiated club cells compared to other club cell markers. We first evaluated and compared the spatial expression pattern of Cldn10 in relationship to CCSP through immuno-histochemical (IHC) double-labeling for CCSP and Cldn10 in the lung sections from IPF and COPD patients. In COPD lungs, CCSP expression was mostly confined to bronchiolar epithelium, wherein CCSP and Cldn10 signals often colocalized in an organized manner (Figure 1A & B). In addition, solely Cldn10-positive cells were occasionally encountered in alveolar walls (Figure 1A). In IPF lungs, bronchiolar epithelium displayed various expression patterns of Cldn10 depending on the size of the bronchioles. In small bronchioles, which were often located in highly fibrotic areas of IPF lungs, solely Cldn10-positive cells were noted to represent a large portion of the bronchiolar epithelium (Figure 1C & D). In the epithelium of large bronchioles of IPF lungs, on the other hand, CCSP signals were sparsely observed in a disorganized pattern (Figure 1E & F), but not in such an organized colocalization pattern with Cldn10 as seen in COPD lungs. In addition some of the club cells positive for CCSP and/or Cldn10 were apparently blended with the fibrotic interstitium (arrows in Figure 1D). Intriguingly, spatial signal transition of club cells from double-positive [CCSP(+) Cldn10 (+)] to solely Cldn10 positive state, was frequently accompanied by the accumulation of elongated cells in the underlying interstitium, which indicates the formation of fibroblastic foci (area circled by dot-dashed line in Figure 1E & F). These results provided us with new insights into the mechanism of epithelial-mesenchymal interaction (EMI) and we hypothesized that when club cells transition from CCSP-positive to CCSP-negative state they activate the underlying fibroblasts. In support of this hypothesis, CCSP-negative Cldn10-positive cells, which are considered to be immature club cells, were frequently found in multiple-layer arrangement near highly fibrotic interstitium (area denoted by arrowheads in Figure 1C).

### Claudin10-positive cells with marked pleomorphism are widely distributed in various patterns in IPF lungs

**Figure 2:**
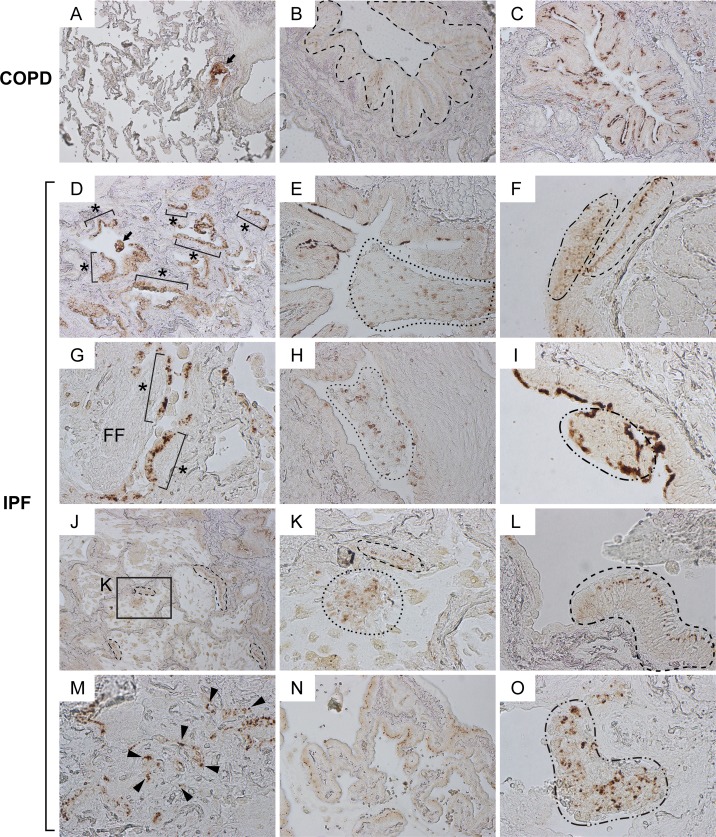
Claudin10-positive cells with marked pleomorphism are widely distributed in various patterns in IPF lungs Paraffin-embedded lung sections from patients with IPF and COPD were immunohistochemically labeled for Cldn10. Brown signals correspond to Cldn10. (**A, B, C**) Representative photomicrographs of normal-looking alveoli with slightly remodeled bronchiolar region (**A**) and bronchiolar regions (**B, C**) from COPD lungs are shown. Despite the variation of subcellular localization of Cldn10, Cldn10-positive cells mostly displayed organized cell arrangements. Arrow in (**A**) denotes cytoplasmic and/or nuclear expression of Cldn10 in the cells at terminal (or respiratory) bronchiole. Dashed-line in (**B**) encompasses bronchiolar epithelium in which Cldn10 signals are confined to the uppermost portion of the lateral membrane between the epithelial cells, apparently corresponding to tight junctions. Cldn10 expression was occasionally spotted at luminal end of the bronchiolar epithelium (**C**). (**D-O**) Representative photomicrographs with different arrangements of Cldn10-positive cells are shown. (**D**) Asterisks denote Cldn10-positive epithelial monolayers in a moderately fibrotic region. Arrow denotes a cellular mass in the airspace containing Cldn10-positive cells. (**E**) Area circled by dotted line denotes mosaic cell mass in the airspace containing Cldn10-positive and negative cells. (**F**) Two different arrangements of club cells were juxtaposed with each other; one with organized columnar cells expressing Cldn10 at the uppermost portion of the lateral membrane between the epithelial cells (area circled by dashed line) and the other with mosaic mixtures of Cldn10-positive and negative cells (area circled by dotdashed line). (**G**) Asterisks denote Cldn10-positive epithelial monolayers. Luminal side of fibroblastic foci (FF) is lined by Cldn10-positive and negative cells with round-to-oval shape. (**H**) Area circled by dotted line denotes a mosaic cell mass containing Cldn10-positive and negative cells. Highly fibrotic area is located to the right of the mass, where Cldn10 signals are barely observed at the luminal edge. **I**: Area circled by dot-dashed line denotes a polypoid cell mass containing Cldn10-positive and negative cells. (**J**) Area with honeycomb change display multiple cysts lined by Cldn10-positive club cell monolayers. (**K**) Magnified view of the boxed region in (**J**) display a mosaic cell mass in the airspace containing Cldn10-positive and negative cells (area surrounded by dotted line) and Cldn10-positive monolayer that partially line the cyst wall (area surrounded by dashed line). (**L**) Area surrounded by dashed line denotes columnar club cells forming organized monolayer. The expression of Cldn10 is confined to the uppermost portion of the lateral membrane between the epithelial cells, apparently at tight junctions. Adjacent bronchiolar epithelium to the left is lined by cuboidal epithelial cells negative or weakly positive for Cldn10. (**M**) Fibrotic interstitium with Cldn10-positive club cells randomly distributed therein. Numerous Cldn10-negative cuboidal cells are also spotted in the vicinity of Cldn10-positive cells. (**N**) Cldn10-positive club cells with oval-to-columnar shape populate the alveolar wall in a monolayer arrangement. (**O**) Area circled by dot-dashed line denotes a mosaic cell mass containing Cldn10-positive and negative cells. The mass barely attaches to the lung structure located to the right. Original magnifications: x100 (**A, J**); x200 (**B, C, D, E, H, N**); x400 (**F, G, I, K, L, M, O**).

To obtain further insights into the potential phenoconversion of club cells in the context of pulmonary fibrosis, we microscopically evaluated immunohistochemically labeled IPF lung sections. In COPD lungs (the control for IPF), Cldn10 expression was largely spotted at cell-to-cell junction sites (area circled by dashed line in Figure 2B) and luminal side of the bronchiolar epithelial cells (Figure 2C). Cldn10 signals were also observed in the cytoplasm and/or nucleus of bronchiolar epithelial cells in terminal and respiratory bronchioles (arrow in Figure 2A) and, occasionally, in alveolar areas (data not shown). In sharp contrast, Cldn10-positive cells in IPF lungs displayed remarkably pleomorphic cell shapes in various arrangements that include, but are not limited to, columnar in monolayer (area circled by dashed line in Figure 2F, J, K & L; Figure 2N), cuboidal in masses (arrow in Figure 2D; areas circled by dotted lines in Figure 2E, H & K; areas circled by dot-dashed lines in Figure 2F & O), cuboidal in monolayer (designated by asterisks in Figure 2D & G), and cuboidal in randomly scattered pattern (arrowheads in Figure 2M). These results suggest that phenoconversion of club cells is actively occurring in a dysregulated manner in IPF lungs.

In order to evaluate the aberrant behavior of club cells in a semi-quantitative manner, two sets of samples (lung sections from IPF and COPD patients; n=6 each) immunohistochemically labeled for Cldn10 were microscopically analyzed using three independent histopathological parameters as follows: (i) hype-rcellularity of Cldn10-positive cells in bronchiolar epithelium, (ii) cellular mass in the airspace containing Cldn10-positive cells, and (iii) infiltration of Cldn10-positive cells into thickened interstitium (Table 1). All of the IPF sections tested (6 out of 6) displayed at least one area with hypercellularity of Cldn10-positive cells (as shown in Figure 2F, I & O) while 17 % of the COPD sections tested (1 out of 6) displayed such a pathologic finding. Cellular masses in the airspace containing Cldn10-positive cells were observed in all IPF sections tested (as shown in areas circled by dotted lines in Figure 2E, H & K) while 50% of the COPD sections tested (3 out of 6) displayed such a pathological finding (as shown in area circled by dotted line in Supplemental Figure S2A). Remarkably, Cldn10-positive cellular masses observed in the airspace of IPF lungs are relatively larger in size and more heterogeneous than COPD. Regarding, infiltration of Cldn10-positive cells into thickened interstitium, 50% of the IPF sections tested (3 out of 6) exhibited at least one area with such a finding (Figure 2M; Supplemental Figure S2B) while such a histopathological finding was not observed in any of the COPD sections tested. Collectively, these results led us to devise plausible cellular events in IPF development, i.e. (1) club cells assume a wide variety of properties depending on their maturity, (2) club cells undertaking varying degrees of phenoconversion proliferate and migrate from bronchiolar to alveolar areas, and (3) a portion of migratory club cells get attached to distant alveoli where they interact with and activate fibro-blasts.

**Table 1:** Histopathological evaluation of abnormal behavior of Cldn10-positive cells in COPD and IPF lungs

	Hypercellularity of Cldn10-positive cells in bronchiolar epithelium	Cellular mass in the airspace containing Cldn10-positive cells	Infiltration of Cldn10-positive cells into thickened interstitium
COPD No. 1	−	−	−
COPD No. 2	−	+	−
COPD No. 3	−	−	−
COPD No. 4	−	+	−
COPD No. 5	+	+	−
COPD No. 6	−	−	−
IPF No. 1	+	+	+
IPF No. 2	+	+	+
IPF No. 3	+	+	−
IPF No. 4	+	+	−
IPF No. 5	+	+	−
IPF No. 6	+	+	+

+: One or more fields exhibit the histopathological finding defined at the top of each column

−: No field exhibits the histopathological finding defined at the top of each column

### Cldn10-positive cells with cuboidal to oval shape line the alveolar epithelium in IPF lungs

**Figure 3:**
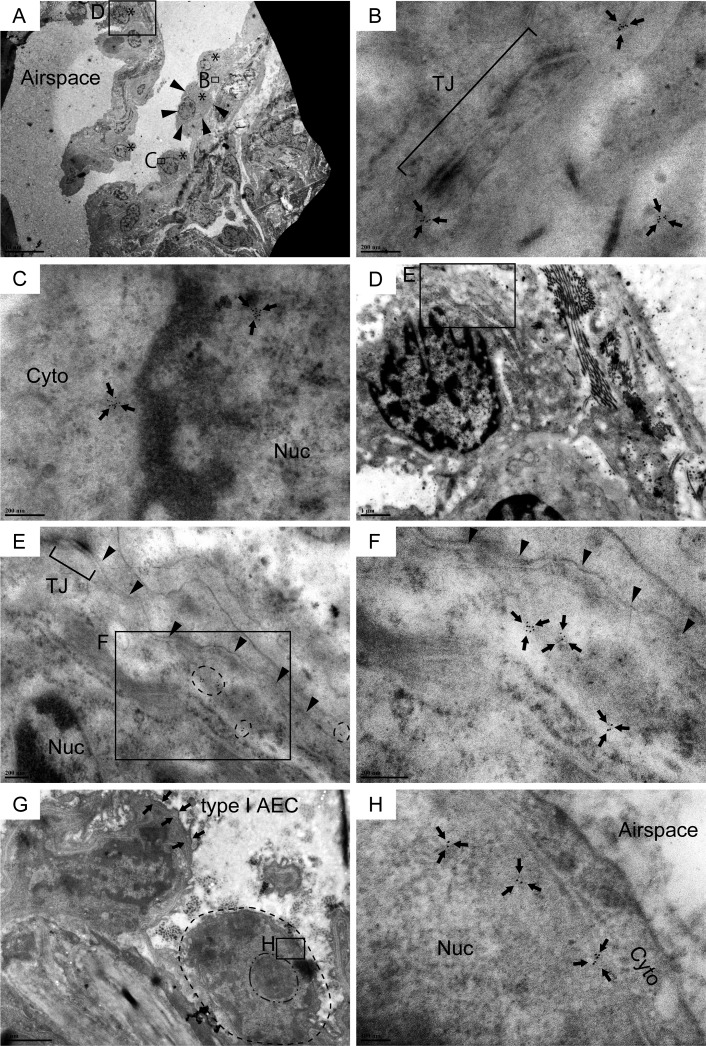
Cldn10-positive cells with cuboidal to oval shape line the alveolar epithelium in IPF lungs Resin-embedded lung sections from patients with IPF were labeled for Cldn10 using immunogold labeling method. Arrows in (**B, C, F** & **H**) denote Cldn10 signals. (**A**) Alveolar epithelium with mild fibrosis is lined by a monolayer of metaplastic epithelial cells with cuboidal to oval shape (Asterisks). (**B**) Cldn10 expression (arrows) are located in close proximity to the tight junction (**TJ**) between the two adjacent cells. Note that the the Cldn10 signals shown belong to the epithelial cell circled by arrowheads in (**A**). (**C**) Cldn10 expression (arrows) are located in the cytoplasm (Cyto) and nucleus (Nuc). Additional photomicrographs showing the spatial association of this cell with the subjacent fibroblast are presented in Supplemental Figure S3F, G & H. (**D, E, F**) Cldn10 expression (tiny black dots in areas circled by dashed lines in (**E**) and arrows in (**F**) are located near the lateral membrane (the boundary between the two adjacent epithelial cells is designated by arrowheads in (**E** & **F**). The Cldn10 signals are not located near to the tight junction (**TJ** in **E**). Note there is a deposition of collagen bundles in the subjacent interstitium of the Cldn10-positive epithelial cell (**D**). (**G, H**) Alveolar epithelium lined by a cuboidal cell with nuclear expression of Cldn10. (**G**) Dot-dashed and dashed line are respectively drawn slightly outside the nucleolus and cytoplasmic membrane of the cuboidal cell. Note that the cell displays large nucleus-to-cytoplasm (N/C) ratio and huge nucleolus. (**H**) Magnified view of the boxed region in (**G**) displays nuclear expression of Cldn10. Original magnifications: x2,000 (**A**); x100,000 (**B, C**); x15,000 (**D**); x60,000 (**E**); x120,000 (**F**); x12,000

**Figure 4:**
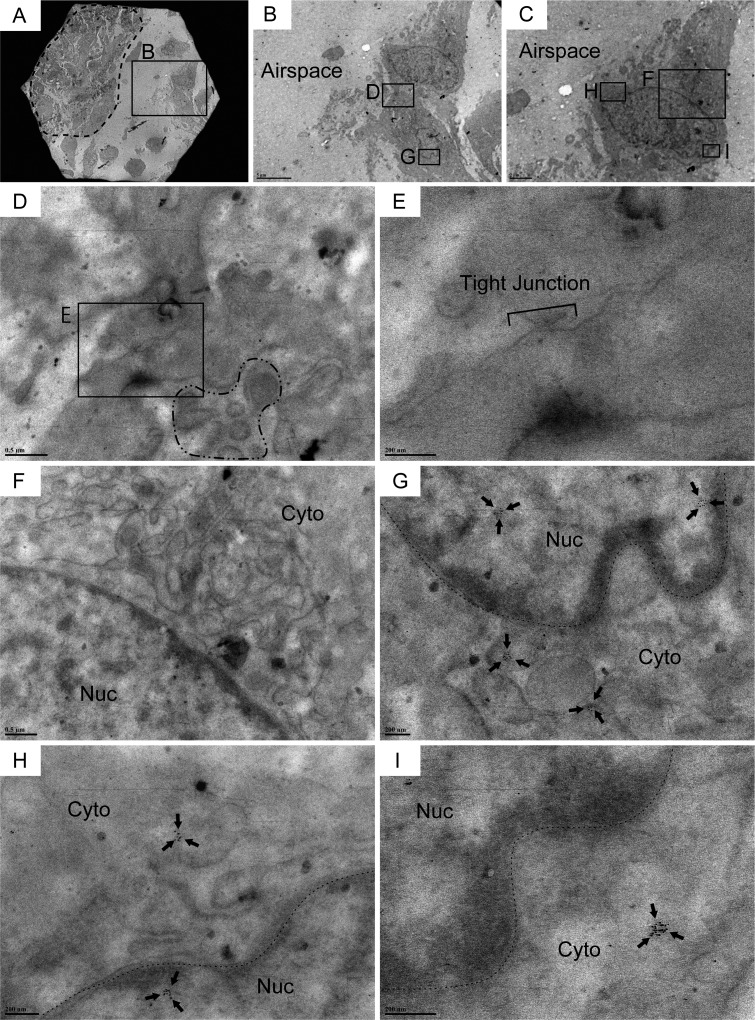
Cldn10-positive cells encompassing traces of subcellular structures of club cells are observed in the airspace of IPF lungs Resin-embedded lung sections from patients with IPF were labeled for Cldn10 using immunogold labeling method. Arrows in (**G, H & I**) denote Cldn10 signals. (**A, B**) Two adjacent cells (within the boxed region in A) are spotted in the airspace off from the fibrotic interstitium to the left (area circled by dashed line in (**A**). Note that the two adjacent cells shown in (**B**) are connected. (**C**) One of the two adjacent cells display elongated oval shape. (**D, E, F**) Two adjacent cells are barely connected at lateral membrane, apparently via tight junction (**E**). Cldn10 is absent in the tight junction (**D** & **E**). Presence of granules in the cytoplasm of one cell is noted (area surrounded by dot-dashed line in (**D**). The other cell displays a well-developed network of endoplasmic reticulum (**F**). (**G**) Magnified view of the boxed region in (**B)** displays cytoplasmic and nuclear expression of Cldn10. (**H, I**) Magnified views of the boxed regions in (**C**) display cytoplasmic (**H, I**) and nuclear (**H**) expression of Cldn10. Original magnifications: x1,500 (**A**); x5,000 (**B**); x8,000 (**C**); x30,000 (**F**); x40,000 (**D**); x60,000 (**G**); x80,000 (**H**); x120,000 (**E, I**).

IHC labeling for Cldn10 using IPF and COPD lung sections revealed that the columnar club cells that form an organized bronchiolar structure often express Cldn10 either at the lateral membrane between adjacent cells or at luminal end of the cells (Figure 2B, C & L) while non-columnar or cuboidal club cells, which often form disorganized spatial arrangement in mainly IPF lungs, apparently express Cldn10 in cytoplasm and/or nucleus (Figure 2A, D, G & O). It can be deduced from these observations that phenoconversion of club cells accompanies subcellular translocation of Cldn10. In other words, subcellular location of Cldn10 is a good indicator of club cell maturity. To confirm that (i) IPF lungs encompass Cldn10-positive club cells that have lost club cell morphology to a certain extent and that (ii) club cells undertaking phenoconversion exhibit cytoplasmic and/or nuclear expression of Cldn10, we probed IPF lung sections for Cldn10 using immunogold labeling method and observed the stained sections under transmission electron microscope (TEM). In con-cordance with the results from IHC staining for Cldn10, relatively organized bronchiolar epithelium comprising columnar epithelial cells often displayed strong Cldn10 signals at the uppermost portion of the lateral membrane (area designated by arrows in Supplemental Figure S3A) as well as their luminal end (area circled by dashed line in Supplemental Figure S3B), even though they usually lack the typical club cell morphology, i.e. dome-shaped luminal surface and cytoplasm filled with secretory granules. In addition, numerous cuboidal to oval cells with varying degrees of Cldn10 expression were spotted in alveolar epithelium as well as in fibrotic interstitium (Figure 3; Supplemental Figure S4). These cuboidal to oval cells, Cldn10 positive and negative, were totally different in appearance from apoptotic cells (Supplemental Figure S3C) and type II AECs spotted in the alveolar area where such cuboidal club cells were found (Supplemental Figure S3D & E). This supports our hypothesis that phenoconversion of adult mature club cells into an immature state involves morphological alteration as well as translocation and associated reduction of Cldn10. The concept of club cell migration was indicated by the presence of Cldn10-positive cells that are spotted in the airspace off the surrounding lung scaffold (Figure 4A, B, C, G, H & I). Cldn10-positive cells found in the airspace of IPF lungs did not show Cldn10 expression at their cell-cell contact sites including tight junctions (Figure 4B, D & E) whereas they displayed slight traces of ultrastructural features of mature club cells, e.g. cytoplasmic granules (area circled by dot-dashed line in Figure 4D) and well-developed endoplasmic reticulum (Figure 4F). Taken together, these results indicate that in IPF lungs, (i) club cells are undertaking active phenoconversion through which they lose typical club cell morphologies seen in mature club cells, (ii) club cells undertaking phenoconversion transmigrate from bronchiolar to alveolar areas and thereby form bronchiolized alveoli and that (iii) phenoconversion of club cells is associated with translocation of Cldn10 from lateral membrane to cytoplasm and nucleus.

### Aberrant α-SMA signals are frequently encountered in the vicinity of disorganized Claudin-10-positive cells in IPF lungs

**Figure 5:**
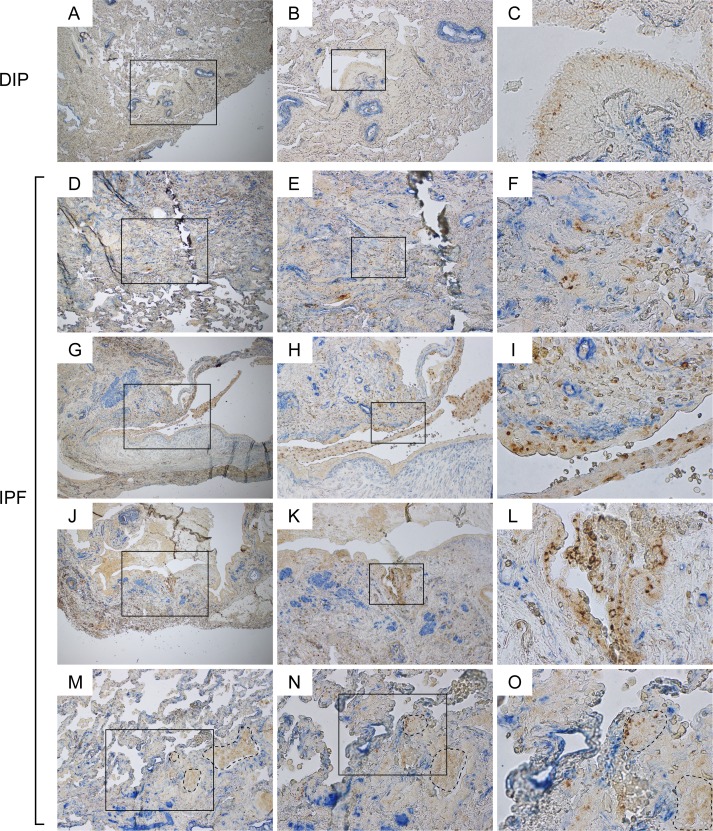
Aberrant α-SMA signals are frequently encountered in the vicinity of disorganized Claudin-10-positive cells in IPF lungs IPF and DIP lung sections were immunohistochemically double-labeled for Cldn10 and ɑ-SMA. Brown and blue signals correspond to Cldn10 and ɑ-SMA respectively. (**B, E, H, K, N**) are magnified views of the boxed regions in (**A, D, G, J, M**) respectively. (**C, F, I, L, O**) are magnified views of the boxed regions in (**B, E, H, K, N**) respectively. (**A, B, C**) Representative photomicrographs from DIP lungs are shown. α-SMA signals are largely spotted beneath the arterial walls, where smooth muscle cells typically reside. No aberrant proximity is seen between Cldn10-positive cells and α-SMA signals. (**D, E, F**) Fibrotic interstitium showing aberrant α-SMA signals in close proximity to Cldn10-positive club cells. Note the juxtaposition of Cldn10-negative cuboidal cells with Cldn10-positive cells and aberrant ɑ-SMA signals (**F**). (**G, H, I**) Mosaic cell masses containing Cldn10-positive and negative cells are floating in an enlarged airspace. Cldn10-positive club cells infiltrating the fibrotic interstitium (**I**). (**J, K, L**) Cldn10-positive club cells form multilayers in bronchiolar epithelium (**L**) in close proximity to intense ɑ-SMA signals (**K**). (**M, N, O**) Boundary region between normal-looking alveoli and moderately affected area with thickened interstitium is shown. Areas surrounded by dashed lines denote mosaic cell masses containing Cldn10-positive and negative cells. Apparently, those cell masses have occluded the alveolar airspaces. Original magnifications: x40 (**A, D, G, J**); x100 (**B, E, H, K, M**); x200 (**N**); x100 (**C, F, I, L, O**).

To further validate the link between Cldn10-positive club cells and IPF pathology, we double labeled lung sections from patients with IPF and desquamative interstitial pneumonia (DIP) (the control for IPF), for Cldn10 and α-SMA, a molecular signature of myofibroblasts. In DIP lungs, the α-SMA signals were largely detected beneath the arterial walls, where smooth muscle cells typically reside, and showed no spatial proximity to Cldn10-positive cells (Figure 5A-C). In contrast, in IPF lungs, Cldn10-positive cells in bronchiolar epithelium and thickened interstitium were frequently juxtaposed with aberrant α-SMA signals (Figure 5D-O). These data, coupled with the aforementioned data suggesting Cldn10-positive cell migration, provide insights into the mechanism of fibrosis initiation in nonfibrotic alveoli in IPF, i.e. a plausible scenario wherein club cells with profibrotic properties randomly migrate and get attached to an alveolar wall, and subsequently interact with and activate the fibroblasts in the subjacent alveolar interstitium.

## DISCUSSION

In the current study, our compelling data suggest that club cell phenoconversion and migration are actively occurring in IPF lungs. Comparison between IPF and control lung sections double-labeled for club cell markers, CCSP and Cldn10, demonstrated relatively well-organized expression pattern of these two markers in control lungs. In contrast, Cldn10-positive club cells in IPF lungs exhibited a wide variety of cell shapes and arrangements in both bronchiolar and alveolar areas. Most importantly, our results indicate that the transition of club cells from CCSP- and Cldn10-positive to a solely Cldn10-positive state in IPF bronchioles, is spatially associated with fibroblastic foci even though it still remains to be elucidated whether such pheno-conversion of club cells is a cause or a result of myofibroblastic phenoconversion of the underlying fibroblasts. Semi-quantitative histopathological evaluation using IPF and control lung sections labeled for Cldn10 led us to develop a new concept that the transition of club cells from a quiescent to active state is associated with their acquisition of proliferative and migratory capacity. Immunogold labeling on IPF lung samples substantiated this concept of “migratory club cells” as a potential trigger for IPF. In addition, having observed a link between Cldn10-positive club cells and aberrant ɑ-SMA signals in IPF lungs, we deduced mechanistic insights into how fibrotic lesions develop and expand in a temporally and spatially heterogeneous manner in IPF. We devised a plausible scenario wherein metaplastic club cells that have acquired proliferative and migratory capacity randomly translocate and eventually attach to nonfibrotic alveoli, and subsequently interact with and phenoconvert interstitial fibroblasts.

Alveolar bronchiolization, a process of proliferation and migration of club cells and other bronchiolar epithelial cell types, has been documented since the 1970s [19]. Its potential relevance to pulmonary fibrosis was club examined in bleomycin-induced lung fibrosis mouse model [11]. Similar phenomena of bronchiolar cell migration to the alveoli were closely examined on paraquat-induced lung fibrosis model in monkeys [9,10]. A systematic lineage-tracing approach supports the notion that migration of club cells is an innate reparative process that is to replace damaged alveoli in the lung [12]. Recently, a novel pathological role for club cells in IPF was proposed, wherein the migratory club cells contribute to IPF progression by promoting epithelial cell death [13]. This concept of migratory club cells as pathogenic participants in IPF is compatible with our observations that Cldn10-positive club cells with morphological pleomorphism, supposedly immature or reverted club cells, are widely distributed in IPF lungs, i.e. in fibroblastic foci (Figure 1E & F; Figure 2G), areas with honeycomb change (Figure 2J & K), and fibrotic interstitium as well (Figure 2M; Figure 5F & I). Certainly, none of the previous studies using microarray analysis reported the upregulation of Cldn10 in IPF. Our preliminary studies using qPCR analysis did not show any significant upregulations of Cldn10 in IPF vs COPD or DIP lungs (data not shown). However, it is clear from our data that translocation of Cldn10, from lateral membrane or luminal edge of the club cells to their cytoplasm and/or nucleus, is closely associated with phenoconversion of club cells (Figure 3C & H; Figure 4G, H & I). So far, no papers have reported the presence of cytoplasmic or nuclear translocation of Cldn10 in any human diseases or cell types. However, nuclear translocation of Cldn2, another member of claudin family, has been implicated in lung cancer and associated with cell proliferation in lung epithelial cells [20]. In melanoma cells, Cldn1 localized in cytoplasm as well as nucleus, with its expression in the former correlating to increased migration [21]. From such examples of other members of the claudin protein family, it can be reasonably deduced that cytoplasmic and/or nuclear translocation of Cldn10 too is associated with a certain type of phenoconversion involving acquisition of proliferative and migratory abilities.

The emergence of metaplastic cuboidal cells is one of the key histopathological features of IPF [22]. Remarkably, the metaplastic epithelial cells overlaying myofibroblasts in fibroblastic foci exhibit cuboidal or oval shapes from our data (Figure 2G) as well as previous report [23]. Kawanami et al. (i) categorized cuboidal epithelial cells seen in fibrotic lung diseases including IPF, based on their subcellular structures under electron microscope observation, into two subtypes, type A and type B, and (ii) surmised, based on their ultrastructural similarities to well-characterized epithelial cell types, that type A and type B cuboidal epithelial cells are derived respectively from bronchiolar basal cells and respiratory bronchioles [22]. Our current study demonstrates that in IPF lungs Cldn10-positive cuboidal or oval cells widely populate fibrotic and nonfibrotic regions in varied arrangements, i.e. as a single cell (Figure 3G & H), in monolayer (areas designated by asterisks in Figure 2D & G; Figure 3) and in massive forms (arrow in Figure 2D; area surrounded by dot-dashed line in Figure 2O). Intriguingly, Cldn10-positive cellular masses were frequently observed in the airspace of IPF lungs and they are usually mosaic mixtures containing Cldn10-positive and negative cells with the latter being predominant (areas circled by dotted lines in Figure 2E & H; areas circled by dot-dashed lines in Figure 2I & O; area circled by dotted line in Figure 2 K; Supplemental Figure S2F, G & H). In addition, Cldn10 ^neg or low^ cuboidal cells were often encountered in overlaying epithelium in fibroblastic foci (Figure 2G; Supplemental Figure S2C & D). One possible explanation for these heterogeneous profiles of metaplastic cuboidal cells in IPF lungs is that they are derived from different cell types, i.e. club cells, and non- club progenitor cells such club as basal cells. This hypothesis is compatible with the previous report by Kawanami et al. wherein 9 out 17 IPF samples tested contain both type A and type B cuboidal epithelial cells [22]. Another plausible explanation for this heterogeneity is “depending on the extent to which club cells dedifferentiate or revert back, their expression of Cldn10, as well as CCSP, decrease in a time-lagged manner (i.e., the decease of CCSP expression is followed by Cldn10 diminution).” This hypothesis is in agreement with our experience obtained through TEM observation wherein (i) IPF lungs encompass club cells with remarkably pleomorphic cell shapes and varying degrees of Cldn10 expression and (ii) clusters of Cldn10-negative cells with high nuclear/cytoplasmic ratio (N/C) and clear chromatin are often found in fibrotic interstitium (Supplemental Figure S4). Intriguingly, Watson et al. demonstrated that deficiency of CCSP causes alteration in club cell ultrastructure and increase in immune cell-specific transcripts during lung remodeling, suggesting that club cell phenoconversion-induced its acquisition of immune cell property and consequent decrease in CCSP secretion plays a pivotal role in lung remodeling [24].

Despite extensive studies that have been performed so far in the field of IPF, it still remains to be elucidated what cellular and molecular events cause perpetual phenoconversion of quiescent fibroblasts into a distinct ɑ-SMA-expressing cell type, i.e. myofibroblasts [23]. Transforming growth factor beta 1 (TGF-β1) is a potent fibrogenic cytokine that plays a crucial role in IPF pathogenesis [25]. One of the most clinically relevant roles of TGF-β1 in IPF is to cause myofibroblastic phenoconversion [26]. In fibroblastic foci, high levels of TGF-β1 are spotted in hyperplastic epithelium overlaying myofibroblasts [27]. Given the fact that a certain proportion of the epithelial cells that overlay myofibroblasts in fibroblastic foci are Cldn10-positive (Figure 2G), it is plausible that dedifferentiated club cells trigger myofibroblastic phenoconversion through TGF-β1 secretion. This hypothesis is consistent with our data showing that in fibrotic areas of IPF lungs, Cldn10-positive cells were requently juxtaposed with aberrant α-SMA signals (Figure 5F, I, L & O). In additional support of this hypothesis, our preliminary studies revealed that oxidative stress, an alleged risk factor for IPF, accelerates emergence of proliferative and TGF-β1-secreting club cells in certain mutant mice (separate manuscript). If such a profibrotic pheno-conversion of club cells is a critical trigger for the appearance of myofibroblasts in IPF, then inhibition of club cell phenoconversion can reasonably be a novel therapeutic option for the treatment of IPF.

Type II AEC abnormality has long been implicated in IPF. Mutations in SFTPC, a representative gene expressed in type II AECs, have been linked to familial pulmonary fibrosis. Additionally, Type II AEC hyperplasia is an established histopathological finding in IPF, indicating the important contribution of type II AECs to IPF pathogenesis. In the present study, we did not perform quantitative or qualitative evaluation of the relative contributions of different epithelial cell types including type II AECs and club cells. However, ourTEM observations revealed that a small number of dysmorphic lamellar bodies are frequently spotted in columnar epithelial cells lining bronchiolar epithelium, and occasionally encountered in metaplastic cuboidal cells in fibrotic interstitium (separate manuscript). These observations imply that in IPF lungs a certain proportion of epithelial cells with pluripotency, including CCSP-negative immature club cells, are preferentially committed to differentiate into type II AECs. If this is the case, pro-SPC-positive metaplastic type II AECs frequently observed in IPF lungs may be derived, at least in part, from club cells or non- club progenitor cells such as basal cells. Thus, it is plausible that the true nature of IPF lies in impaired differentiation of lung progenitor cells into normal and functional type II AECs.

Lineage tracing method using cell type-specific transgenic mice has provided access to otherwise unattainable information, especially regarding the cell types that participate in the development of pulmonary fibrosis [12,28,29]. Unfortunately, cell-specific markers can diminish upon cell differentiation or dedifferen-tiation, which makes it difficult to trace highly plastic cell lineage. Club cells have been known to change their profiles in response to environmental stressors [8], and our data also indicate that club cells are actively changing their epithelial properties in IPF lungs (Figure 1E & F). Further, while tracing of CCSP-positive club cells have long been available to researchers, a mutant strain enabling Cldn10 tracing is not yet available. Therefore, we reached a notion that relating subcellular ultrastructures of metaplastic epithelial cells to club cell markers in IPF lungs would provide us with otherwise unattainable information as to the origin of metaplastic lung epithelial cells. As expected, our studies have given us mechanistic insights about the origin of metaplastic cuboidal cells in IPF.

Little information is available on how club cells senesce during ageing, especially in the context of pulmonary fibrosis. Zhou et al. proposed that club cell senescence plays an important role in COPD pathogenesis by revealing a higher percentage of club cells in COPD lungs that are positive for CCSP as well as p16, a representative marker of ageing, compared to control lungs from asymptomatic nonsmokers [30]. They related senescent club cells to impaired alveolar regeneration and exacerbated inflammation in COPD. On the other hand, our data suggest that in IPF lungs club cell acquisition of proliferative as well as migratory ability plays a key role in impaired alveolar regeneration and consequent chronic fibrosis. It may well be that the balance between age-related club cell senescence and its survival adaptation determines the fate of the aged lungs.

**Figure 6:**
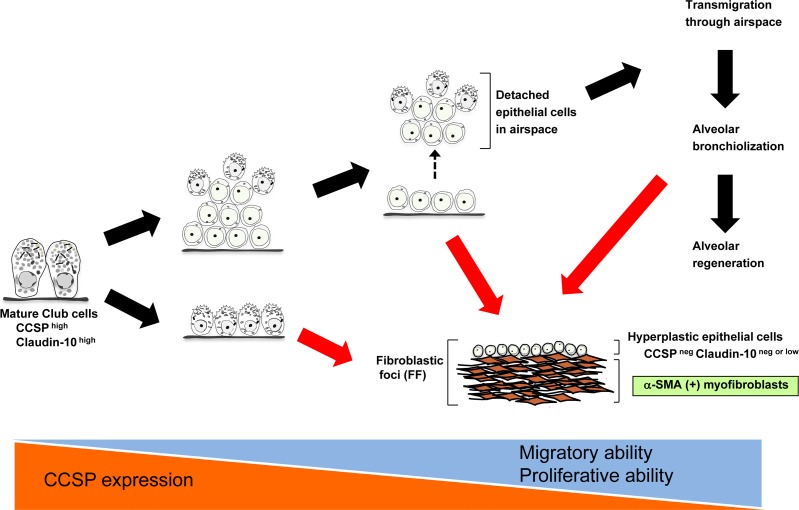
A hypothesized model of club cell involvement in IPF pathogenesis Black arrows denote putative reparative cascades by club cells. Red arrows denote our hypothesis regarding how club cell phenoconversion leads to the formation of fibroblastic foci (FF) in IPF development.

In conclusion, our new approach combining immuno-histochemical double staining and immunogold staining has allowed us to propose a new paradigm where club cells actively participate in IPF pathogenesis from early stages through perpetual phenoconversion (summarized proposal is shown in Figure 6). This new concept does not necessarily conflict with the current central paradigm of IPF where type II AECs play a critical role in IPF pathogenesis assuming that club cells under stressed conditions can lose their properties and gain type II AEC character as an innate reparative program. Further investigation is required to validate this new hypothesis. If the aberrant pheno-conversion of club cells and impaired differentiation of Club cells into functional type II AECs are established as essential triggers for myofibroblastic phenoconversion of normal fibroblasts, then club cell-targeted therapy might become one of the powerful strategic options for the treatment of IPF.

## MATERIALS AND METHODS

### Human IPF, COPD, and DIP samples

Human lung samples from patients with IPF, patients with chronic obstructive pulmonary disease (COPD), patients with decreased lung function and histological diagnosis of emphysema (added to the category of “COPD”), and patients with desquamative interstitial pneumonia (DIP) were obtained from the Lung Tissue Research Consortium (LTRC) funded by the National Institutes of Health (NIH). Formalin-fixed paraffin-embedded (FFPE) lung tissue sections from patients with IPF, COPD, or DIP were used for immunohisto-chemical staining. Glutaldehyde-fixed lung samples from patients with IPF were used for immunogold labeling.

### Single-color immunohistochemical staining on lung tissue sections

Single-color immunohistochemical (IHC) staining was performed on paraffin-embedded lung tissue sections as follows. First, paraffin sections were deparaffinized. Then, sections were subjected to heat-induced antigen retrieval (HIAR) in a citric acid buffer (10 mM citric acid, pH 6) or Tris-EDTA buffer (10 mM Tris, 1mM EDTA, 0.05% Tween 20, pH 9.0). Then, endogenous peroxidase activity was quenched with 3% hydrogen peroxide in PBS for 20 min. Subsequently sections were blocked with PBS containing 5% bovine serum albumin (BSA) plus 0.25% Tween 20, and sequentially with 10% goat serum (diluted in PBS). Sections were then incubated with rabbit anti-Cldn10 antibody (Life Technologies, Carlsbad, CA) at 4°C overnight. After washing, the sections were incubated for 30 min with goat anti-rabbit IgG antibody, which is conjugated with horseradish peroxidase (HRP) (Histofine Simple Stain Mouse MAX Peroxidase; Nichirei, Tokyo, Japan). Finally, detection of the target protein was performed using 3, 3′-diaminobenzidine (DAB) reagent.

### Double-color immunohistochemical staining on lung tissue sections

Deparaffinization, antigen retrieval, and quenching of the endogenous peroxidase activity were performed in the same way as in single-color IHC staining. Subsequently, the sections were blocked with PBS containing 5% BSA plus 0.25% Tween 20. The sections were incubated at 4°C overnight with either goat anti-CCSP antibody (Santa Cruz Biotechnologies, Santa Cruz, CA) or rabbit anti-Cldn10 antibody (Life Technologies, Carlsbad, CA) (1st round primary antibody). After washing, the sections were incubated respectively with donkey anti-goat IgG antibody conjugated with HRP (Thermo Fisher Scientific, Waltham, MA) or goat anti-rabbit IgG antibody conjugated with HRP (Histofine Simple Stain MAX Peroxidase). Detection of the target protein was performed using DAB reagent. After washing with distilled water and PBS, sections were heated at 90°C for 10 min in citric acid buffer (10 mM citric acid, pH 6) in order to abrogate the enzymatic activity of the HRP remaining on the sections. The tissue sections were blocked with PBS containing 5% BSA plus 0.25% Tween 20. Then, they were incubated respectively with rabbit anti-Cldn10 antibody or mouse anti-alpha smooth muscle actin (ɑ-SMA) antibody (2nd round primary antibody). After overnight incubation at 4°C with the primary antibody, tissue sections were washed and incubated respectively with either goat anti-rabbit IgG antibody conjugated with HRP (Histofine Simple Stain MAX Peroxidase) or goat anti-mouse IgG antibody conjugated with alkaline phosphatase (ALP) (Sigma-Aldrich, St. Louis, MO). Detection of the target protein was respectively performed using PolyDetector HRP Green Kit (Bio SB, Santa Barbara, CA) or Vector Blue Alkaline Phosphatase (ALP) substrate kit (Vector Laboratories, Burlingame, CA).

### Immunogold labeling and transmission electron microscopy

IPF lung samples (2-4 mm in diameter) fixed in glutaraldehyde, were delivered from the Lung Tissue Research Consortium (LTRC) (n=5). After being received, they were cut into smaller pieces and re-fixed with one of the following two methods: (1) 2% glutaraldehyde in sodium cacodylate containing 0.5% tanic acid or (2) 0.5% reduced osmium in sodium cacodylate. Following overnight fixation, the samples were rinsed in cacodylate buffer. Then, only samples fixed in 2% glutaraldehyde containing 0.5% tanic acid were further fixed in a mixture of 0.75% osmium tetroxide and 0.025M imidazole in 0.1M cacodylate buffer (pH 7.5) for 30 min at 37°C. All fixed samples were then treated with 0.5% uranyl acetate en bloc at room temperature for 2 hrs, followed by dehydration in graded ethanol and acetone. The dehydrated lung samples were processed for epoxy resin embedding using EMbed-812 (Electron Microscopy Sciences, Hatfield, PA, USA). Thin sections (90-100 nm) were cut using an ultramicrotome (UCT; Leica, Wetzlar, Germany). The sections were then treated with 4% aqueous solution of sodium metaperiodate for 15 min. After washing in water, the sections were treated for 15 min with 1% teleostean gelatin, 0.1% BSA, 10 mM HEPES, 150 mM NaCl, and 0.1% Tween-20 in PBS (referred to as teleostean gelatin-containing buffer hereinafter). Then, the sections were incubated at 4°C for overnight with rabbit anti-Cldn10 antibody (Life Technologies) in 1% telestean gelatin-containing buffer. After washing once in teleostean gelatin-containing buffer, the sections were incubated at room temperature for 30 min with goat anti-rabbit IgG antibody conjugated with gold particle (Cat. #25364, Electron Microscopy Sciences, Fort Washington, PA). After washing 10 times in distilled water, the sections were dried out at room temperature. The prepared samples were examined at 80 kV using a transmission electron microscope (JEM 1400; JEOL, Tokyo, Japan) equipped with a digital camera (Gatan, Inc., Pleasanton, CA). Note, as negative controls, sections treated with primary antibody but without secondary antibody and sections without primary antibody but with secondary antibody were prepared.

### Semi-quantitative histological evaluation of the aberrant behavior of Cldn10-positive cells

Randomly chosen paraffin-embedded lung sections from IPF and COPD patients (1 section per 1 patient; n=6 for each group) were immunohistochemically labeled for Cldn10 utilizing the single-color staining method and DAB as described above. The presence or absence of three independent histopathological findings was microscopically evaluated for each section. The three parameters include i) hypercellularity of Cldn10-positive cells in bronchiolar epithelium, ii) cellular mass in the airspace containing Cldn10-positive cells, and iii) infiltration of Cldn10-positive cells into thickened interstitium.

## SUPPLEMENTARY MATERIAL FIGURES


