# Supplementary material for: The role of club cell phenoconversion and migration in idiopathic pulmonary fibrosis
Source: Aging (Albany NY). 2016 Nov 29;8(11):3091–105. doi: 10.18632/aging.101115 (PMC5191887; doi:10.18632/aging.101115)
Supplement: Supplementary file 1 [file aging-08-3091-s001.pdf]

SUPPLEMENTARY MATERIAL

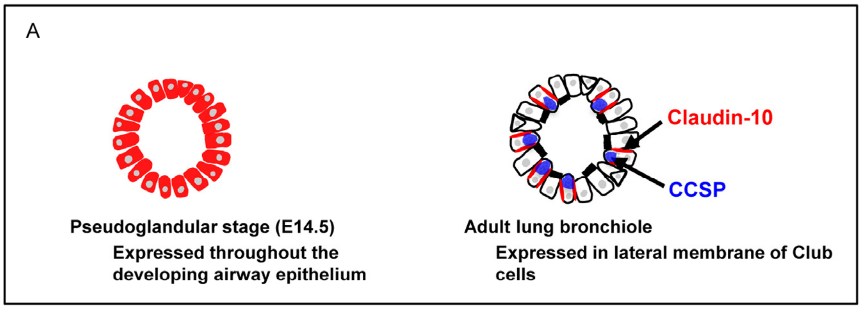

Adapted with permission of American Thoracic Society from Zemke et al. Am J Respir Cell Mol Biol 2009, 40:340-348.
Copyright © 2016 American Thoracic Society.

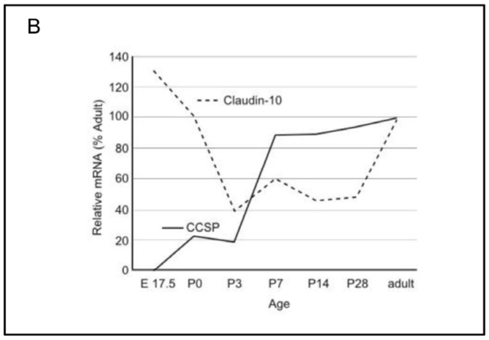

Adapted with permission of American Thoracic Society from Zemke et al. Am J Respir Cell Mol Biol 2009, 40:340-348.
Copyright © 2016 American Thoracic Society.

Supplemental Figure S1. Temporal and spatial expressions of Cldn10 and CCSP in developing and adult mouse lungs. The figures shown are adapted from a previous report by Zemke et al. with permission of the American Thoracic Society. Copyright © 2016 American Thoracic Society. Author: Zemke et al, Title: Molecular Staging of Epithelial Maturation Using Secretory Cell–Specific Genes as Markers, Journal title: Am J Respir Cell Mol Biol, Year: 2009, Vol 40 Pages 340-348. The American Journal of Respiratory Cell and Molecular Biology is an official journal of the American Thoracic Society. Claudin-10 (Cldn10; shown in red in **(A)** is a protein whose expression in the early developing lungs is observed throughout the premature bronchiolar epithelium (left in (**A**) and converges to the entire lateral surface of club cells (right in (**A**) as they mature and begin to express club cell secretory protein (CCSP; shown in blue in (**A**). The expression of Cldn10 in mouse lungs reaches adult expression levels as early as embryonic day (E) 17.5, an earlier time-point than CCSP, of which expression at E17.5 is still very low and only reaches adult expression levels as late as postnatal day 7 (P7) (**B**).

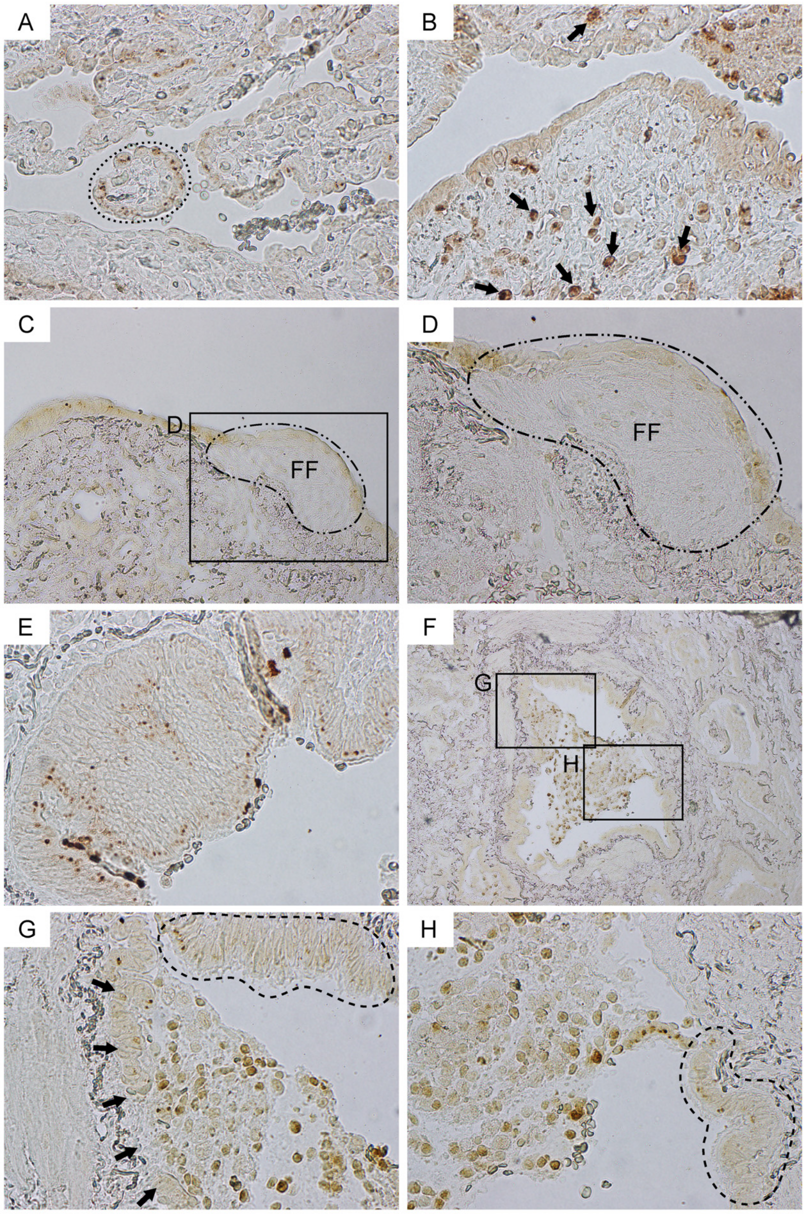

Supplemental Figure S2. Cldn10 expression in COPD and IPF lungs. Paraffin-embedded lung sections from COPD (**A**) and IPF (**B-H**) patients were immunohistochemically labeled for Cldn10. Brown signals denote Cldn10. (**A**) A remodeled area from COPD lungs is shown. Dotted-line denotes cellular mass in the airspace containing Cldn10-positive and negative cells. (**B**) Fibrotic interstitium is infiltrated by Cldn10-positive cuboidal cells (arrows). (**C, D**) Epithelial cells lining fibrotic regions exhibit gradual transition from columnar to cuboidal cells depending on the proximity to fibroblastic foci (**FF**). Cldn10 expression decreases in parallel with the morphological transition from columnar to cuboidal shape. (**E**) Hyperplastic bronchiolar epithelium comprising cuboidal cells negative and positive for Cldn10. (**F, G, H**) Mosaic cellular mass containing Cldn10-positive and negative cells occupy a large portion of the bronchiolar lumen (**F**). Areas circled by dashed lines in (**G**) and (**H**) denote columnar epithelium with no discernible atypia. Top-left of the cellular mass is barely attached to the bronchiolar epithelium located to the left, wherein the epithelial cells are cuboidal to oval in shape (arrows in **G**). Original magnifications: x400 (**A, B, D, E, G, H**); x200 (**C**); x100 (**F**).

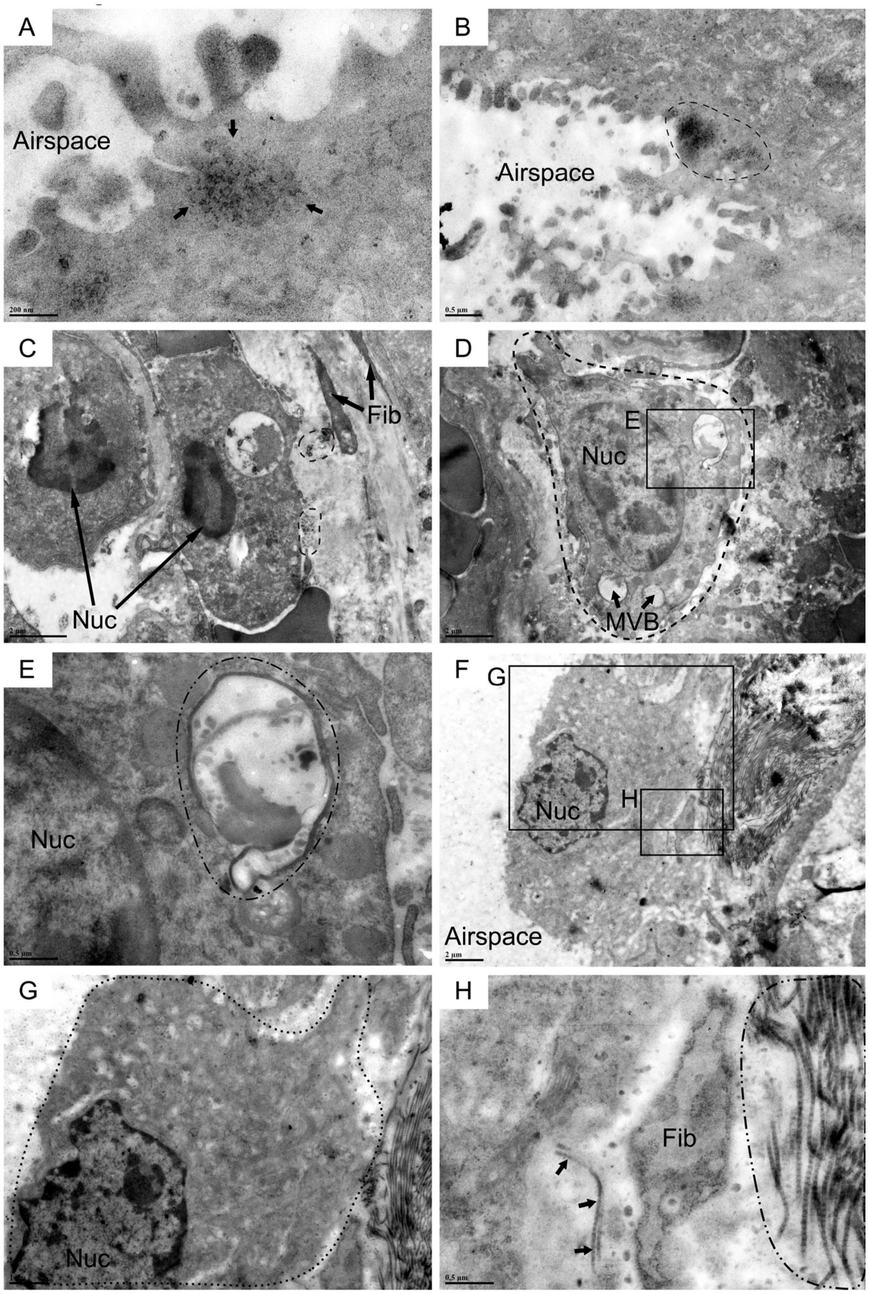

Supplemental Figure S3. Cldn10-expressing epithelial cells in bronchiolar and alveolar epithelium of IPF lungs. Resin-embedded lung sections from patients with IPF were labeled for Cldn10 using immunogold labeling method. (**A, B**) Columnar bronchiolar epithelium in IPF lungs exhibits Cldn10 signals at the uppermost portion of the cell-cell contact site (area circled by arrows in (**A**) and at the luminal edge of the plasma membrane (area circled by dashed-line in (**B**). **C:** Two apoptotic cells with condensed chromatin, a major morphological feature of apoptosis, are located adjacent to loose connective tissue. Apparently apoptotic fibroblasts (Fib) and collagen bundles (areas circled by dashed lines) are seen in the connective tissue. (**D, E**) Type II alveolar epithelial cell (area circled by dashed line in (**D**) featuring multivesicular bodies (MVB) and a MVB with slight traces of lamellar bodies (area circled by dot-dashed line in (**E**), is spotted in the vicinity of Cldn10-positive cuboidal cell (Figure 3G & H**)**. (**F, G, H**) Cldn10-expressing metaplastic epithelial cell (circled by dotted line in **G**; refer to Figure 3A & B since this cell is the same cell as circled by arrowheads in Figure 3A) forming close contact with the subjacent fibroblast (Fib in **H**). Collagen bundles to the right of the fibroblast are designated by dot-dashed line (**H**). Collagen fibers are observed between the adjacent epithelial cell and fibroblast (arrows in **H**), indicating a close interaction between them. Original magnifications: x30,000 (**A**); x100,000 (**B**); 12,000 (**C**); x10,000 (**D**); x40,000 (**E, H**); x8,000 (**F**); x15,000 (**G**).

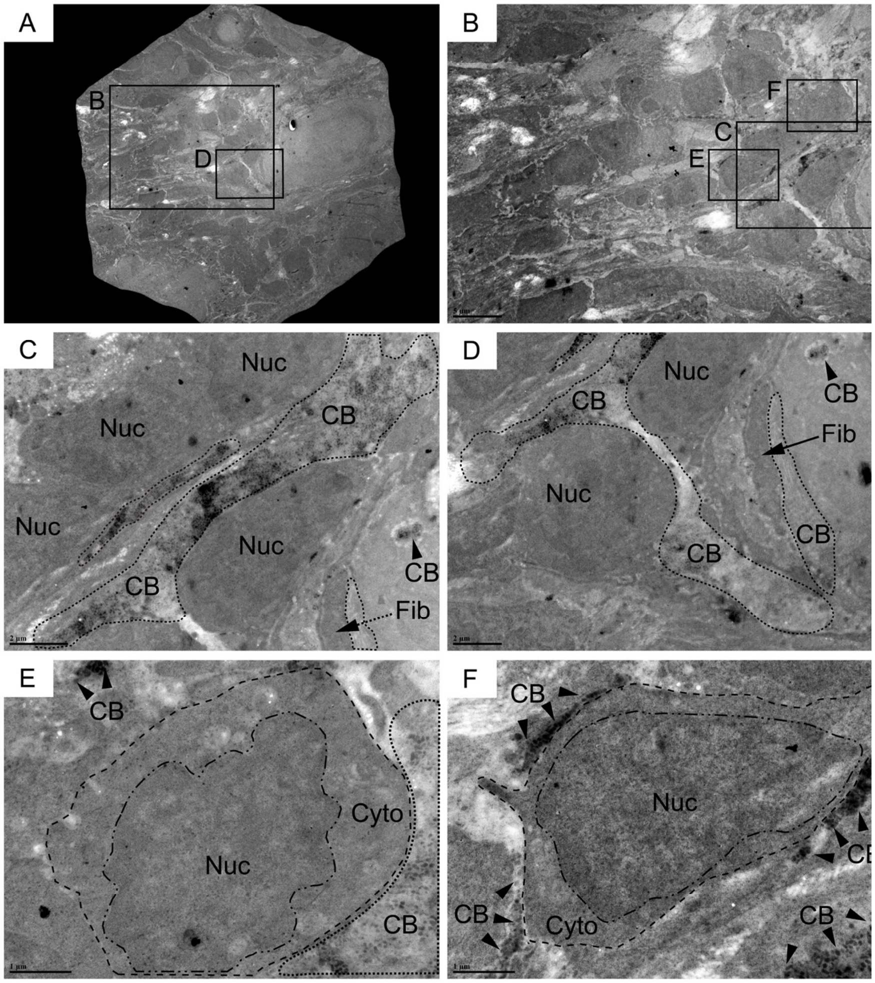

Supplemental Figure S4. Cuboidal cells with high nuclear/cytoplasmic (N/C) ratio showing a cluster arrangement in IPF lung. Resin-embedded lung sections from patients with IPF were labeled for Cldn10 using immunogold labeling method. (**A, B, C, D, E, F**) Fibrotic area with specific arrangement of cells and connective tissues featuring cluster-forming Cldn10-negative cuboidal cells with high N/C ratio (**E, F**), adjacent fibroblasts (Fib in (**C** & **D**), and in-between collagen bundles (CB)**;** circled by dotted lines)**.** Original magnifications: x1,500 (**A**); x4,000 (**B**); 12,000 (**C**); x10,000 (**D**); x25,000. (**E, F**) Dot-dashed and dashed lines are respectively drawn slightly outside the nucleolus and cytoplasmic membrane of the cuboidal cells.
